# Fabrication and characterization of well-aligned and ultra-sharp silicon nanotip array

**DOI:** 10.1186/1556-276X-7-120

**Published:** 2012-02-13

**Authors:** Chi-Chang Wu, Keng-Liang Ou, Ching-Li Tseng

**Affiliations:** 1 Graduate Institute of Biomedical Materials and Tissue Engineering, Taipei Medical University, Taipei, 11031, Taiwan; 2 Research Center for Biomedical Devices, Taipei Medical University, Taipei, 11031, Taiwan

**Keywords:** silicon nanotip, well-aligned, field emission, Fowler-Nordheim, oxide-capped

## Abstract

Well-defined, uniform, and large-area nanoscaled tips are of great interest for scanning probe microscopy and high-efficiency field emission. An ultra-sharp nanotip causes higher electrical field and, hence, improves the emission current. In this paper, a large-area and well-aligned ultra-sharp nanotip arrays by reactive ion etching and oxidation techniques are fabricated. The apex of nanotips can be further sharpened to reach 3-nm radius by subsequent oxidation and etching process. A schematic model to explain the formation of nanotip array is proposed. When increasing the etching time, the photoresist on top of the nanotip is also consumed, and the exposed silicon substrate is etched away to form the nanotip. At the end, the photoresist is consumed completely and a nanotip with pyramid-like shape is developed. The field emission property was measured, and the turn-on field and work function of the ultra-sharp nanotip was about 5.37 V/μm and 4.59 eV, respectively. A nanotip with an oxide layer capped on the sidewall is also fabricated in this paper. Comparing to the uncapped nanotip, the oxide-capped sample exhibits stable and excellent field emission property against environmental disturbance.

## Background

Ultra-sharp nanotips are drawn a lot of attention because of their promising applications in many fields such as electronics, microscopy, nanolithography, and biology [1-3]. More recently, application of nanotip in high-efficiency field emission [FE], flat panel displays, scanning probe microscopy, and scanning tunneling microscopy are intensively investigated [4]. The efficiency of these techniques strongly depends on the characterization of the tip. For instance, a high-brightness and quick-response FE display can be obtained by a well-aligned nanotip array. FE is a quantum phenomenon that electrons are emitted from the cathode by a large electric field and tunneled through the surface of the tip array. This technique is a potential method for the manufacture of high-quality display with features of thin panel thickness, wide view angle, low power consumption, and high tolerance. Generally, the tip-end size, as well as the arrangement of the nanotip array, plays an important role in performing an effective FE [5]. A tip with smaller apex can induce higher electrical field and, hence, significantly enhances the emission current [6,7].

Various materials are employed to form the nanotips [8-10]. Among them, silicon is one of the most promising candidates to fabricate nanotips because it is the most used materials and ease of fabrication in the micro-electronic field [11-13]. Numerous technologies have been developed recently for the preparation of silicon nanotips, including evaporation deposition, electroplating, anisotropic wet etching method, and dry etching method [14-18]. Huang et al. used porous anodic alumina membrane as the mask and obtained a Si nanotip array by removing the silicon oxide [SiO_2_] islands which were formed during anodization of the Al/Si interface [14]; Cheng et al. fabricated silicon nanotips through high-density hydrogen plasma etching [15]; Hsu et al. proposed a one-step and self-masked dry etching technique for fabricating uniform and high-single-crystal silicon nanotips [16]. These methods demonstrate simple processes to fabricate ultra-sharp nanotips. The alignment of nanotip array is, however, unsolvable issue because the nanotips are formed randomly by these methods. Linn et al. developed a microfabrication-compatible technology using inverted silicon pyramidal pits to fabricate the periodic gold nanopyramids with nanoscale sharp tips [18]. Using this method, aligned silicon tip is obtained, but it is difficult to fabricate nanotips with high aspect ratio and sharp end since only pyramidal shape structures are provided.

In this work, Si nanotip arrays by combining the photolithography and reactive ion etching technology are fabricated. The apex of the nanotip can reach down to 3 nm in radius. By using this method, a large-area, well-aligned, and patternable nanotip array with high aspect ratio, ultra-sharp tip end can be achieved. In addition, we propose a formation scenario and model to explain the experiment results. The FE properties of Si nanotip arrays are investigated. The results indicate that the FE properties of nanotip arrays are improved when sharpening the tip end by oxidation process. We also demonstrate an oxide-capped nanotip which is only tip-end exposed. The oxide-capped sample exhibits stable and excellent field emission property against environmental disturbance.

## Methods

### Fabrication of the nanotip

The ultra-sharp nanotip array was fabricated using a commercially available 6-in., (100)-oriented, p-type silicon wafers. After standard (NH_4_OH/H_2_O_2 _= 3:1, then HCl/H_2_O_2 _= 3:1) and hydrofluoric acid [HF] cleaning, a 700-nm-thick photoresist [PR] was coated using TEL Clean Track Model-MK8 system (Tokyo Electron Limited, Tokyo, Japan), followed by 300 × 300 nm^2 ^square array was defined using the optical exposure (Canon FPA-3000i5 stepper, Canon, Tokyo, Japan) systems. The plasma etching system (Lam Research TCP9400SE, Lam Research Corporation, Fremont, CA, USA) was then employed to form the pyramid-like tips. The etching power and pressure were 300 W and 12 mTorr, respectively. The gas flow was Cl_2_/HBr = 35:125 sccm.

To further sharpen the tip, a SiO_2 _was thermally grown to oxidize the sidewall of the tips, and then, samples were immersed into a buffer oxide etch [BOE] (NH_4_F/HF = 6:1) solution to fully remove the silicon oxide.

### Fabrication of oxide-capped nanotip

**Figure 1:**
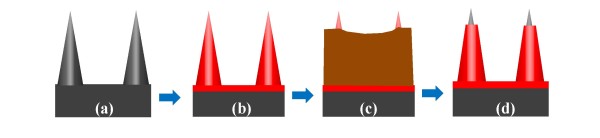
**Schematic representation of formation of the oxide-capped nanotips**. (**a**) Original silicon nanotips. (**b**) A 30-nm-thick silicon oxide was thermally grown. (**c**) Photoresist was spin-coated and immersed into the stripper to expose the pinpoint of nanotips. (**d**) The oxide-capped nanotips were completed.

The schematic representation of fabrication steps of the oxide-capped nanotip is provided in Figure 1. Firstly, a 50-nm-thick SiO_2 _film was thermally grown using a furnace system (Figure 1b), and a PR was then spin-coated onto the surface. To match the height of the nanotip, we tuned the rotation rate of the spin coating to decrease the PR thickness. After PR coating, the samples were immersed into the PR stripper for 3 sec, and hence, the pinpoint of nanotip was exposed, as shown in Figure 1c. Then, samples were immersed into the BOE solution to remove SiO_2 _on the pinpoint surface. Finally, the PR was then removed by H_2_SO_4 _and H_2_O_2 _mixture, and the nanotips with an oxide capping layer on the sidewall was completed (Figure 1d).

### Property analysis of the nanotip

Microstructure of the nanotip array was examined using SEM. The field emission characteristics were measured at 1E-6 Torr using a Keithley 237 high-voltage semiconductor parameter analyzer (Keithley Instruments, Inc., Cleveland, OH, USA). The silicon nanotips served as the lower electrode, and the tungsten probe approached the nanotips gradually to 100 nm. The tungsten probe was then applied a positive voltage ranging from 0 to 1,000 V and measured the emission current.

## Results and discussion

The progress of transformation of the tips and photoresist at different etching time is displayed in Table 1. From the eagle-view and top-view scanning electron microscope [SEM] images, it can be clearly seen that the size of PR is reducing upon the etching time. Silicon beneath the PR is then exposed and etched away. Therefore, the shape of the silicon underneath is gradually transformed from pillar to tip since the sidewall of the silicon pillar is pared by etchant. Finally, the PR is consumed completely after 10-min etching time, and a pyramid-like tip is then formed. The appearance of the pyramid-like tip shows sharp apexes with 12 nm in radius and 500 nm in height. To further sharpen the tip-end radius, the pyramid-like tip was treated to a thermal oxidization process. After the sharpening process, the apex of nanotip can be reached down to 3 nm in radius while retaining almost the same height.

**Table 1:** Eagle-view and top-view SEM images of the tips at different etching time

	Etching time				
	3 min	4 min	6 min	10 min	After sharpening
Tip images					
Top view					

Shape conversion of the tip at various etching times can be observed by top-view images. The shape of the nanotip significantly influences the field emission property. Interestingly, the exterior appearance of the tip is transforming with etching time. As seen in Table 1, the top-view appearance of the initial PR is rectangular, while after 4-min etching time, the tip is turning into round-corner pyramid shape; at 6-min etching time, the corner of the pyramid shape becomes acute; after 10-min etching time, the tip transforms to recessed pyramid shape. Although the mechanism needs to be further studied, this phenomenon is imaginable from the point of view of etch probability.

**Figure 2:**
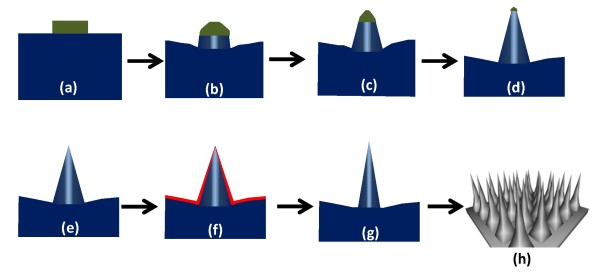
**Formation model of the ultra-sharp nanotip array**. (**a**) Photoresist coated and patterned on the silicon substrate (**b**) after 3-min etching, (**c**) after 4-min etching, and (**d**) after 6-min etching. (**e**) After 10-min etching, a pyramid-like tip was formed. (**f**) A 50-nm-thick silicon oxide was thermally grown. (**g**) After silicon oxide removal, an ultra-sharp nanotip is formed. (**h**) A 3-D schematic representation of the ultra-sharp silicon nanotip array.

Based on the observation results in Table 1, we propose a formation model of the ultra-sharp nanotip array. Figure 2 shows the schematic representation of nanotip formation at different etching times. The coated PR on top of the tips serves as the shielding mask to protect the underlying silicon (Figure 2a). The etching depth of silicon is increased upon etching time, while the PR is also gradually etched away; the exposed silicon is then increased and etched away. Therefore, the sidewall of the silicon pillar is pared and transformed into tip (Figure 2b, c, d). Finally, the PR is fully etched away after 10-min etching, and a pyramid-like tip shape is formed (Figure 2e). To further sharpen the tip, a thermal oxidation and wet etching process is performed (Figure 2f), and a high aspect ratio, ultra-sharp nanotip array can be achieved, as shown in Figure 2g, h.

**Figure 3:**
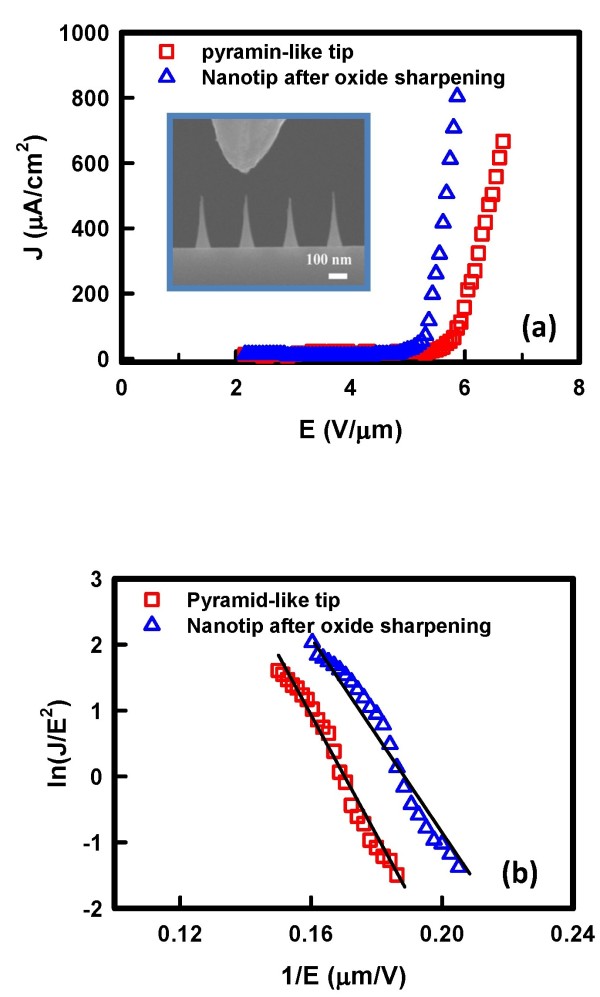
**Field emission properties of silicon tips**. (**a**) Current density-electrical field curves of silicon tips. Inset shows the SEM image of the nanotip array during field emission measurement. The top electrode is tungsten probe. (**b**) Fowler-Nordheim plot of silicon tips.

The FE characteristics of nanotips were measured at room temperature. Figure 3a shows the typical FE curves of the pyramid-like tip and sharpened nanotip. Inset of Figure 3a displays the real-time SEM images of the nanotip array during field emission measurement. One can see that the tungsten probe on top of the nanotip served as the top electrode to apply a voltage. The obtained J-E curves demonstrate that the sharpened nanotip exhibits improved FE property than the pyramid-like tip. The turn-on fields are 5.92 and 5.37 V/μm for the pyramid-like tip and sharpened nanotip, respectively.

To analyze the FE properties of nanotip array, Fowler-Nordheim [FN] law is used to describe the relationship between current density and the local electrical field. The FN law is expressed by the following equation [19]:

(1) $$\ln\left(\frac{J}{E^{2}}\right)=\frac{-B\emptyset^{\Xi /2}\beta^{-1}}{E}+\ln\left(A\emptyset^{-1}\beta^{2}\right)$$

where A and B are constants equal to 1.56 × 10^-10 ^A eV V^-2 ^and 6.83 × 10^3 ^eV^-3/2 ^V μm^-1^, respectively. The field enhancement factor is *β*, and *ϕ *is the work function; *β *and *ϕ *could be extracted by fitting the straight line from the ln(J/E^2^) versus 1/E plot. Figure 3b illustrates the FN plot of the pyramid-like tip and sharpened nanotip. The FN plots show a linear relationship, implying that the quantum tunneling effect is the main mechanism for the FE. The extracted field enhancement factors from the FN plots are 711 and 818 for the pyramid-like tip and sharpened nanotip, respectively.

**Figure 4:**
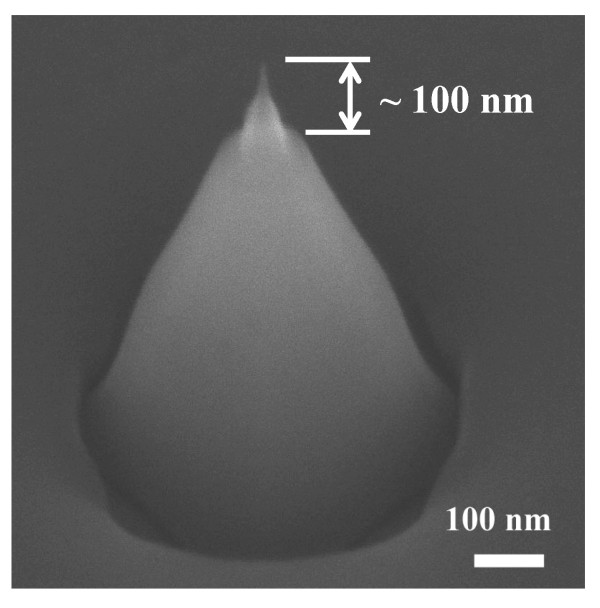
**SEM image of the oxide-capped nanotip**. The exposed tip end is about 100 nm.

**Figure 5:**
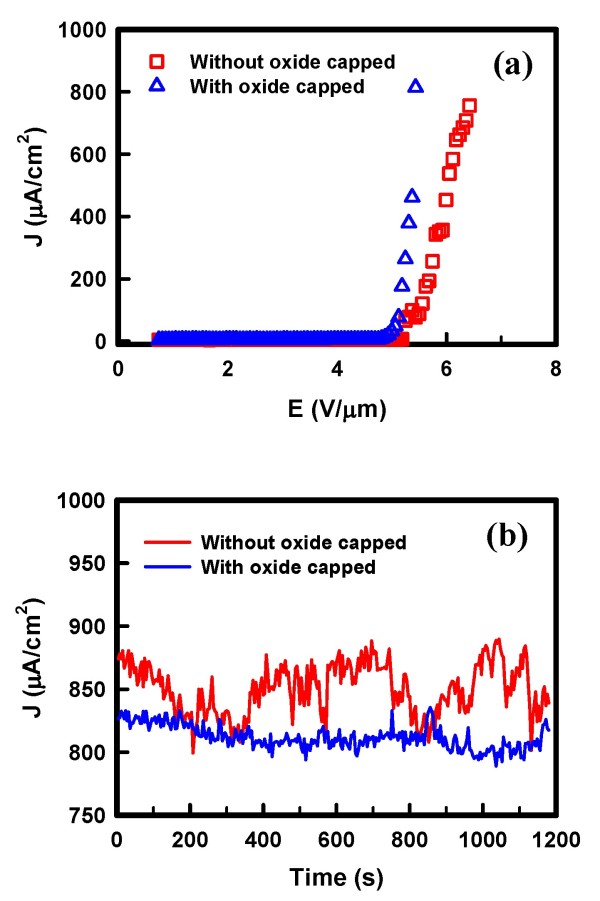
**Field emission properties of the uncapped and oxide-capped nanotips in disturbance environment**. (**a**) Current density-electrical field curves of nanotips. (**b**) Current density-time of nanotips.

To avoid the current noise from environmental disturbance, an oxide-capped nanotip was also fabricated. The oxide-capped nanotip exhibits a silicon oxide film capped on the sidewall of the tip, while only the pinpoint of the nanotip is exposed. The SEM images of oxide-capped nanotip are shown in Figure 4. The exposed pinpoint is about 30 nm. Figure 5 shows the FE properties when the nanotips are in contaminated environment. We dropped a droplet to the nanotip which served as the disturbance environment and measured the J-E curves. As shown in Figure 5a, the FE property of uncapped nanotip exhibits disturbance in turn-on current, indicating that the uncapped nanotip is disturbed by the contamination. On the other hand, the current of the oxide-capped nanotip retains a sharp curve. Furthermore, the turn-on field of the oxide-capped sample is slightly improved compared with the uncapped one. Figure 5b shows the plot of emission current versus time for the nanotips in the disturbance environment. The fluctuation of the uncapped nanotip is 20%, while the oxide capped sample is 9.4%. Obviously, the stability is making better for the oxide capped nanotip. This result demonstrates that the oxide capping layer is effective as the prevention of the environmental disturbance, and the FE property is improved when applying the voltage.

## Conclusions

We have fabricated a large-area and well-aligned ultra-sharp nanotip array by photolithography and reactive ion etching techniques. The apex of the nanotip can reach to 3 nm in radius. The mechanism of nanotip formation is that the remained photoresist on top of the tip is gradually consumed during the etching process, and the exposed silicon substrate is etched away to form the nanotip. The field emission property of the ultra-sharp nanotip is measured, and the turn-on field and work function of the ultra-sharp nanotip was estimated about 5.37 V/μm and 4.59 eV, respectively. The oxide-capped nanotip was also fabricated and demonstrated its excellent property against contamination.

## Competing interests

The authors declare that they have no competing interests.

## Authors' contributions

CCW and CLT carried out the experiment and measurement. CCW prepared the manuscript. KLO technically supported the study. All authors read and approved the final manuscript.
